# A Systematic Review of Myocarditis Induced by Immune Checkpoint Inhibitors: How Concerning Is the Most Common Cardiotoxicity of Immune Checkpoint Inhibitors?

**DOI:** 10.7759/cureus.42071

**Published:** 2023-07-18

**Authors:** Ali Moradi, Athri Kodali, Chiugo Okoye, Dhadon Hannah Klein, Iman Mohamoud, Olawale O Olanisa, Panah Parab, Priti Chaudhary, Sonia Mukhtar, Lubna Mohammed

**Affiliations:** 1 Internal Medicine, California Institute of Behavioral Neurosciences & Psychology, Fairfield, USA

**Keywords:** immune check-point inhibitors, immune check point inhibitor adverse effects, pembrolizumab side effect, late cardiotoxicity, ipilimumab nivolumab, nivolumab-related adverse events, fulminant myocarditis, immune therapy-mediated myocarditis, immune therapy mediated myocarditis, immune check-point inhibitor

## Abstract

Novel cancer therapies have revolutionized the management of various cancers. An immune checkpoint inhibitor (ICI) is one of these antitumor medications. ICIs, which are immune therapies, enhance the immune system's capacity to fight cancer cells. Based on the receptors that they inhibit, such as PD-1, PD-L1, and CTLA-4, ICIs are subdivided. Although this class of drugs is extremely beneficial for cancer patients, their adverse effects can be fatal. Multiple organs, such as the cardiovascular system, may be impacted by immune-related adverse effects (irAEs). These cardiotoxic irAEs can occur at a rate of up to 1% and can be fatal. Myocarditis is the most prevalent of all cardiotoxicities. The purpose of this systematic review is to assess the seriousness of myocarditis, the most prevalent cardiotoxicity of ICIs, and the importance of screening. We chose studies based on the Preferred Reporting Items for Systematic Reviews and Meta-Analyses (PRISMA) 2020 criteria. Therefore, from 2018 to 2023, we gathered articles from databases such as PubMed, ScienceDirect, Web of Science, the Cochrane Library, and Google Scholar. Of the 665 studies identified based on various screening methods and quality assessment tools, 13 were selected for inclusion in the study. This study shows that although the risk of myocarditis in ICI therapy is low and the majority of cases are asymptomatic or mild, some cases can be deadly and disastrous, and physicians should be aware that if myocarditis is suspected based on clinical symptoms, troponin, electrocardiogram, and echocardiogram, treatment should be initiated accordingly.

## Introduction and background

Previous oncology research studies have considerably aided in the development of high-expectancy treatments, allowing physicians to combat cancer much more effectively than in the past. These investigations aided in the development of anti-tumor medications that considerably improved life expectancy and reduced deaths. Although life expectancy is growing, this also causes to see more long-term side effects such as cardiotoxicity [1]. Tumor cells can avoid immune surveillance by activating checkpoint pathways. Using this mechanism, tumor cells can create an unlimited number of neoplastic cells. Immune checkpoint inhibitors (ICIs) are a type of anti-tumor medicine. The development of ICI in immunological oncology was a significant achievement in cancer therapy [2]. ICIs are immunotherapies designed to assist the immune system in its fight against tumors. The way they work is by binding to immunological receptors on T cells. Unlike traditional cancer treatments, ICIs focus on enhancing the ability of immune cells to target cancerous cells. ICIs balance the inflammatory and anti-inflammatory responses. Checkpoint inhibitors can be inhibitory or stimulatory in nature. There are various types of antibodies in this category that are responsible for targeting receptors, such as CTLA-4, PD-1, and PD-L [3]. One of the drug groups in ICIs is PD-1 and PD-L1, and PD-L1 is a ligand for PD-1. Since PD-L1 is present in a variety of cancers, many researchers have studied this group, and their research has yielded encouraging results in cancers like head and neck squamous cell carcinoma [4]. As PD-1 inhibitors, the US FDA has approved three monoclonal antibodies: nivolumab, pembrolizumab, and cemiplimab. Avelumab and durvalumab are two PD-L1 inhibitors drug [3]. T cells must attach to CD80 in order to activate; however, CTLA-4 has the ability to inhibit this binding. CD28 and CTLA-4 both attach to CD80 on antigen-presenting cells (APC), but CTLA-4 has a higher affinity for binding to CD80, which inhibits T cell activation CTLA-4 inhibition promotes T cell activation. Ipilimumab, one of the drugs in this group, has shown promising results in cancer therapy. Ipilimumab was the first medicine to demonstrate positive results in metastatic melanoma in comparison to dacarbazine-based chemotherapy, which shows five years of survival in 20% of patients [5]. Although ICIs have many advantages for oncology patients, they also have a wide range of side effects on various organs such as the cardiopulmonary system, skin, gastrointestinal tract, nervous, hematologic, ocular systems and etc. However, they differ from the adverse effects of traditional cancer therapies such as chemotherapies. Although ICIs can be used with minor immune-related adverse effects (irAEs), we must be cautious of potentially fatal irAEs [6]. irAEs in ICIs are typically caused by non-specific immune system activation, and they can result in the discontinuation of treatment in around 40% of patients [7]. irAEs are often treated with high-dose glucocorticoids; nevertheless, in some situations, persistent and severe episodes can occur [8]. Cardiotoxicity is one of the irAEs of ICIs; despite all, it is rare, with a 1% incidence, but it is frequently severe and alarming [9]. Although cardiotoxicity is mentioned at 1%, total cardiac events are at 3.1% and even up to 9.7% in other studies [10,11]. However, the gap between 3.1% and 9.7% can be influenced by covid-19 myocarditis, which can be confused with ICIs myocarditis [12]. Numerous forms of cardiotoxicity irAEs, including myocarditis, pericarditis, Takoto cardiomyopathy, arrhythmias, conduction disorders, and myocardial infarction, have been linked to the use of ICIs. The most prevalent, however, is myocarditis [9]. The cardiotoxic effect of ICIs is one of many factors that can contribute to myocarditis, an inflammation of the heart muscle. The mortality of myocarditis in patients treated with ICIs can be up to 50% [9]. Myocarditis as an irAEs after starting ICIs can occur as soon as two weeks; however, the median period for the event to occur is 65 days [13,14]. In this study, we review previously published articles to look into various types of immune checkpoint inhibitors and the most common cardiotoxicity caused by ICIs, myocarditis. Despite numerous reviews on the subject, minimal research has been conducted to determine whether myocarditis caused by ICIs is of concern. In this systematic review, we aim to summarize information about patients who acquired myocarditis due to ICIs to evaluate if it is more fatal than we thought.

## Review

Method

This systematic review followed the Preferred Reporting Items for Systematic Reviews and Meta-Analyses (PRISMA) 2020 guidelines [15]. A literature search was conducted by the first author and co-authors utilizing databases including PubMed, ScienceDirect, Web of Science, Cochrane Library, and Google Scholar. The search took place using keywords including “cardiotoxicity,” “myocarditis,” “checkpoint inhibitors,” “Nivolumab,” “pembrolizumab,” “CTLA-4 inhibitor,” “PD-1 inhibitor,” and “PL-L1 inhibitors” and combining them using the BOOLEANs “AND” and “OR.” A mesh strategy was employed to reduce the number of published articles. Table 1 provides a summary of the databases examined for article collections and the respective search strategy.

**Table 1:** Databases that are used to collect articles.

Search strategy	Database used	Number of research papers identified
immune checkpoint inhibitors and cardiotoxicity	ScienceDirect	112
((("Cardiotoxicity"[Mesh]) AND ( "Cardiotoxicity/diagnosis"[Mesh] OR "Cardiotoxicity/drug therapy"[Mesh] OR "Cardiotoxicity/etiology"[Mesh] OR "Cardiotoxicity/immunology"[Mesh] OR "Cardiotoxicity/mortality"[Mesh] OR "Cardiotoxicity/pathology"[Mesh] OR "Cardiotoxicity/physiopathology"[Mesh] ) AND ( "Immune Checkpoint Inhibitors/administration and dosage"[Mesh] OR "Immune Checkpoint Inhibitors/adverse effects"[Mesh] OR "Immune Checkpoint Inhibitors/blood"[Mesh] OR "Immune Checkpoint Inhibitors/pharmacology"[Mesh] OR "Immune Checkpoint Inhibitors/poisoning"[Mesh] OR "Immune Checkpoint Inhibitors/therapeutic use"[Mesh] OR "Immune Checkpoint Inhibitors/toxicity"[Mesh] ) ) OR ((cardiotoxicity)) AND (checkpoint inhibitor))	PubMed	340
immune checkpoint inhibitors and cardiotoxicity	Google Scholar	110
immune checkpoint inhibitors and cardiotoxicity	Cochrane Library	2
immune checkpoint inhibitors and cardiotoxicity	Web of Science	88

Eligibility Requirement

Studies were selected for inclusion based on the following criteria related to participants, interventions, and outcomes. Participants in this study are cancer patients receiving immune checkpoint inhibitors for cancer therapy, and the outcomes are patients who had the most prevalent cardiotoxicity caused by ICI.

Inclusion and Exclusion Criteria

In addition to the above inclusion criteria, only papers written in English, published between 2018 and 2023, and focusing on human studies have been considered for the review. In this review, various kinds of immune checkpoint inhibitors were employed; however, only myocarditis, the most common form of cardiotoxicity, was considered; other forms of cardiotoxicity were excluded. The paper contains populations of both men and women. In the study, only full-text articles are included. The study excluded book chapters, conference papers, abstracts, gray literature, and animal research.

Results

**Figure 1:**
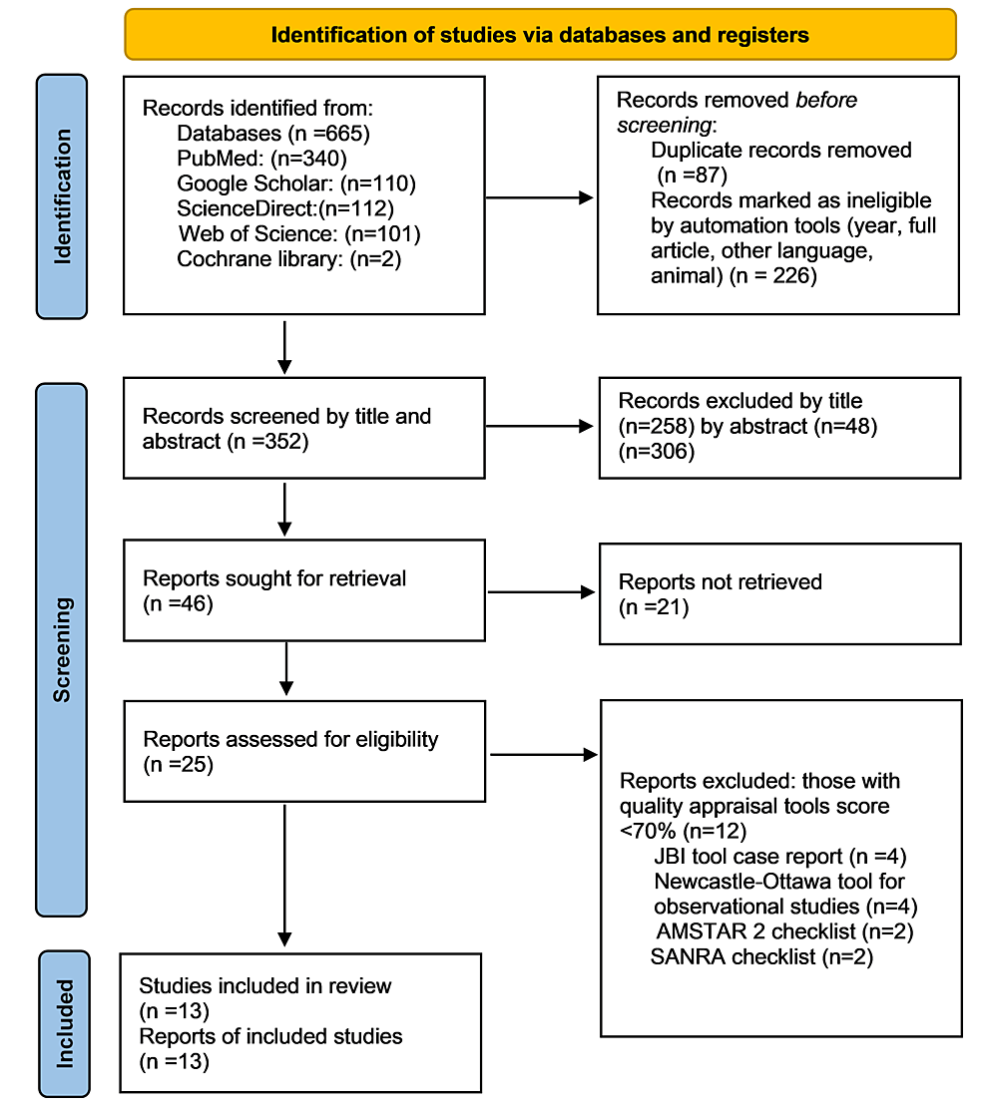
The PRISMA chart, an overview of the screening procedure. AMSTAR 2: Assessment of Multiple Systematic Reviews 2, SANRA: Scale for the Quality Assessment of Narrative Review Articles, JBI Critical Appraisal Checklist: Joanna Briggs Institute Critical Appraisal Checklist, PRISMA: Preferred Reporting Items for Systematic Reviews and Meta-Analyses [15].

Using different databases with the mentioned search strategies, a total of 665 records were extracted from the databases, and different inclusion criteria filters were used to exclude 226 studies. In the next step, 87 duplicate articles were recognized and removed. A total of 352 articles were screened based on title and abstract, 46 studies were selected to investigate more, and 25 studies were subjected to quality assessment by using quality assessment tools such as the JBI quality assessment checklist (for case reports), the Newcastle-Ottawa quality assessment checklist (for case-control and cohort studies), AMSTAR 2 (for systematic reviews and meta-analyses), and SANRA (for narrative review articles), and a final number of 13 were selected to include in the study. The PRISMA chart, shown in Figure 1, gives an overview of the screening process [15].

The last database search for this systematic review occurred on May 2, 2023. Table 2 summarizes the final studies that were included after the quality assessment.

**Table 2:** Summary of all studies, include in this systematic review. SANRA checklist accepted score (>=70%): Minimum score 9 out of 12; Newcastle Ottawa tool accepted score (>=70%): Minimum score 6 out of 9, AMSTAR 2 checklist accepted score (>=70%): Minimum score 12 out of 16, JBI Critical Appraisal Checklist accepted score (>=70%): Minimum score 6 out of 8; AMSTAR 2: Assessment of Multiple Systematic Reviews 2, SANRA: Scale for the Quality Assessment of Narrative Review Articles, JBI Critical Appraisal Checklist: Joanna Briggs Institute Critical Appraisal Checklist.

Author	Study type	Assessment tool	Score
Asuka Furukawa et al. 2023 [16]	Prospective study	Newcastle-Ottawa tool	8
Zomorska et al. 2022 [17]	Case study	JBI critical appraisal checklist	6
Chuan Zhang et al. 2022 [18]	Case study	JBI critical appraisal checklist	7
Andrew T.Nguyen et al. 2022 [19]	Case study	JBI critical appraisal checklist	6
Danielle Delombaerde et al. 2022 [20]	Case study	JBI critical appraisal checklist	7
Yasuyuki Miyauchi et al. 2021 [21]	Case study	JBI critical appraisal checklist	7
Ravi A. Thakker et al. 2021 [22]	Review article	SANRA	10
Shiying Liu et al. 2020 [23]	Case study	JBI critical appraisal checklist	7
Grigorios Chatzantonis et al. 2020 [24]	Case study	JBI critical appraisal checklist	7
Samantha Champion et al. 2020 [25]	Retrospective study	Newcastle-Ottawa tool	7
Atallah-Yunes et al. 2019 [26]	Systematic review	AMSTAR 2	13
Syed S. Mahmood et al. 2018 [27]	A retrospective and prospective study	Newcastle-Ottawa tool	7
Sarju Ganatra et al. 2018 [28]	Case study	JBI critical appraisal checklist	7

Table 3 displays a summary and characteristics of the case reports included in this study, including the age and gender of the patients, the type of cancer they had, the type of ICI used to treat cancer, clinical symptoms, the day after the onset of symptoms, treatment, and outcomes.

**Table 3:** Characteristics and different aspects of case reports on myocarditis induced by ICIs. ICI = Immune checkpoint inhibitor, F = Female, M = Male, SCC = Squamous cell carcinoma. Table created by the first author, Ali Moradi.

Case	Age/sex	ICI	Malignancy	Day of symptom presentation after the last ICI	Presenting symptoms	Treatment	Outcome
Zomorska et al. 2022 [17]	59/F	Nivolumab	Squamous cell thymic carcinoma	14 days after	Dyspnea	Supportive + immunosuppressant	The patient died after six days
Chuan Zhang et al. 2022 [18]	69/M	Camrelizumab	Hepatocarcinoma	One week after	Shortness of breath	Supportive + diuretic + corticosteroid	The patient died three weeks after
Chuan Zhang et al. 2022 [19]	75/M	Camrelizumab	Bronchial SCC3	Three weeks after	Numbness, muscle weakness, blurred vision, shortness of breath	Corticosteroid	The patient’s condition improves after two weeks
Andrew T. Nguyen et al. 2022 [20]	48/M	Pembrolizumab	Non-small cell lung cancer	Nine months after	Fever, nausea, vomiting, chest pain	Corticosteroid	The patient died on the same day of admission
Danielle Delombaerde et al. 2022 [21]	69/M	Ipilimumab + nivolumab	cholangiocarcinoma	Before 3^rd^ dose, due to abnormal troponin level	Epigastric pain	Aspirin + Tinzaparin	The patient is alive and monitored every two weeks
Yasuyuki Miyauchi et al. 2021 [21]	71/M	Ipilimumab + nivolumab	Renal cell carcinoma	Seven days after 2^nd^ cycle	Chest tightness and shortness of breath	Corticosteroid admitted to the intensive care unit	Despite the cardiogenic shock and ventricular fibrillation, the patient improved.
Shiying Liu et al. 2020 [23]	78/M	Pembrolizumab	Melanoma	Two weeks after 2^nd^ dose	Dyspnea and dysphagia	Corticosteroid+ amiodarone for arrhythmia + abatacept + plasmapheresis	The patient improved and was discharged from the hospital after four months
Grigorios Chatzantonis et al. 2020 [24]	30/F	Pembrolizumab	Pulmonary adenocarcinoma	Suddenly after 6^th^ cycle	Shortness of breath, dyspnea, and a sign of heart failure	Intensive care unit admission, supportive treatment, temporal pacemaker, corticosteroid	Patient improved
Sarju Ganatra et al. 2018 [28]	41/F	Ipilimumab + nivolumab	Metastatic melanoma	Six days after the last cycle (4^th^ cycle)	Mild dyspnea	Corticosteroid	The patient improved, and screening after four months showed improvement

In Table 4, the results and conclusions of observational studies that are included in a systematic review are summarized.

**Table 4:** Summary of observational studies. ICI = Immune checkpoint inhibitor, MACE = Major adverse cardiac events, irAEs = Immune-related adverse effects.

Study	Type of study	Period of study	Result	Conclusion
Asuka Furukawa et al. 2023 [16]	Prospective study	From April 2017 to May 2020	One hundred twenty-six patients with cancer enrolled to study for ICI treatment; 18 (14.3%) had elevated troponin I levels; 13 (10.3%) had symptoms; and 4 (3.2%) needed to stop receiving ICIs.	The study revealed a higher-than-anticipated incidence of myocarditis, but most cases lacked symptoms or were mild enough to continue treatment.
Syed S. Mahmood et al. 2018 [27]	A retrospective and prospective study	From November 2013 to July 2017	Thirty-five patients with ICI-associated myocarditis compared with a control group of 105 patients who were treated with ICIs without myocarditis; the prevalence of myocarditis was 1.14%; 54% didn't have any other irAEs. Most myocarditis was seen in combined ICIs therapy (34% vs. 2%) and diabetes patients (34% vs. 13%). The steroid was administered to 89% of patients.	Myocarditis isn't a common side effect of ICIs, but it's concerning as it can cause major adverse cardiac events (MACE). Due to this fact, the workup and management of myocarditis induced by ICIs are important.
Samantha N. Champion et al. 2020 [25]	Retrospective study	From January 2016 to February 2019	Ten cases of ICI-induced myocarditis were identified (nine based on endometrial biopsy and one based on autopsy). Eight of them had clear signs of myocarditis, and two of them had borderline characteristics of myocarditis. Low-grade ICI myocarditis compared with high-grade ICIs, and there was no significant difference in troponin levels, number of cycles, or even time from the first cycle, although all high-grade ICI myocarditis died even despite therapy.	ICI myocarditis can be high-grade or low-grade. A high-grade ICI has more clinical symptoms and is more aggressive, but a low-grade ICI has mild clinical symptoms. ICIs myocarditis, compared to acute cellular rejection, has more lymphohistiocytic inflammation.

Discussion

Immune checkpoint inhibitors (ICIs) can have a variety of adverse effects, but in this study, we focused on the most prevalent cardiotoxicity, myocarditis [3]. Although there is a difference in the rate of myocarditis caused by ICIs between men and women, some studies have shown that the rate is higher among men, and the average patient age is 68. Among all co-morbidities, hypertension was the most common among patients. Melanoma was the most common cancer associated with myocarditis associated with ICI use, and nivolumab was the most frequently employed ICI [22]. The nivolumab drug database has shown that among the 20,000 patients who were treated with this drug, 0.09% developed myocarditis [8]. However, this only reports symptomatic patients, and perhaps the rate of myocarditis is higher [29]. The most common treatment used among patients with myocarditis-induced ICIs was corticosteroids such as prednisolone and methylprednisolone. However, despite therapy among patients with myocarditis, about half of them died, which is concerning and brought to our attention [22]. In this study's discussion section, we examine the pathophysiology, diagnosis, and treatment of ICI-induced myocarditis.

Pathophysiology

**Figure 2:**
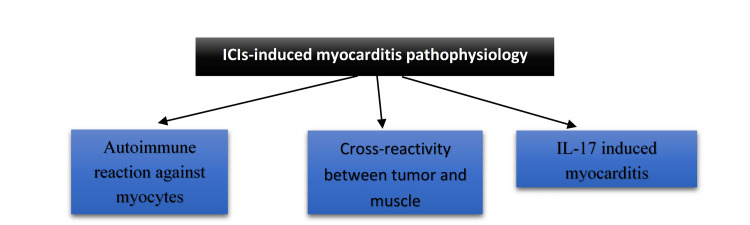
Three potential pathophysiologies behind the myocarditis induced by ICIs. ICI: Immune checkpoint inhibitor, IL: Interleukin. Figure created by the first author, Ali Morad.

Normal immune systems combat cancer cells, but some tumor cells are able to evade the immune system. Immune checkpoint inhibitors (ICIs), however, enable the immune system to fight back against this mechanism. One of the groups of these drugs is PD-1 inhibitors, which are monoclonal antibodies such as nivolumab, pembrolizumab, and cemiplimab. The second group of these drugs is CTLA-4 inhibitors, including ipilimumab, which is the first FDA-approved ICI [22]. A combination of these two groups, specifically ipilimumab and nivolumab, is used to fight cancer, and some studies report myocarditis with this combination [20-22,28]. The pathophysiology of myocarditis-related ICIs is still under research, as the inflammation can cause severe problems such as heart failure as well as other major cardiac events with a high rate of mortality. There are three mechanisms that could potentially be the cause of myocarditis related to ICIs (Figure 2). The first is the autoimmune reaction against myocytes, which can be induced by ICIs' immune system trigger; the second is the cross-reactivity between tumor and cardiac muscle antigen; and the last potential mechanism is that ICIs can increase the level of interleukin (IL)-17, which can be linked to myocyte damage [30]. There are three possible pathophysiologies for ICI-induced myocarditis, which are shown in Figure 2.

Previous studies on mice have shown that when CTLA-4 receptors are out of cells, it can have an effect on myocardial cells and cause fulminant myocarditis. The same experience was seen with PD-1 receptors, showing spontaneous myocarditis. The study also showed a decrease in the level of IL-10, which is an anti-inflammatory cytokine, when PD-1 is absent [26]. Other studies suggest that the rapid onset of myocarditis is most likely due to the role of preexisting memory T cells that increase their activity while PD-1 is blocked by ICIs [29].

Diagnosis

Obtaining a complete cardiac profile is necessary for the diagnosis of myocarditis caused by ICIs. However, common symptoms associated with myocarditis, physical findings, and lab results, which are explained in Table 5, can guide us toward the diagnosis [22].

**Table 5:** Common findings in ICI-induced myocarditis. ICI: Immune checkpoint inhibitor, S3: Third heart sound.

Symptoms	Physical exam	Lab result
Angina	S3 gallop	High troponin
Dyspnea	Jugular venous distension	High brain natriuretic peptide (BNP)
Orthopnea	Lower extremity pitting edema	
Lower extremity edema	Tachycardia	
	Hypotension with cardiogenic shock	

**Figure 3:**
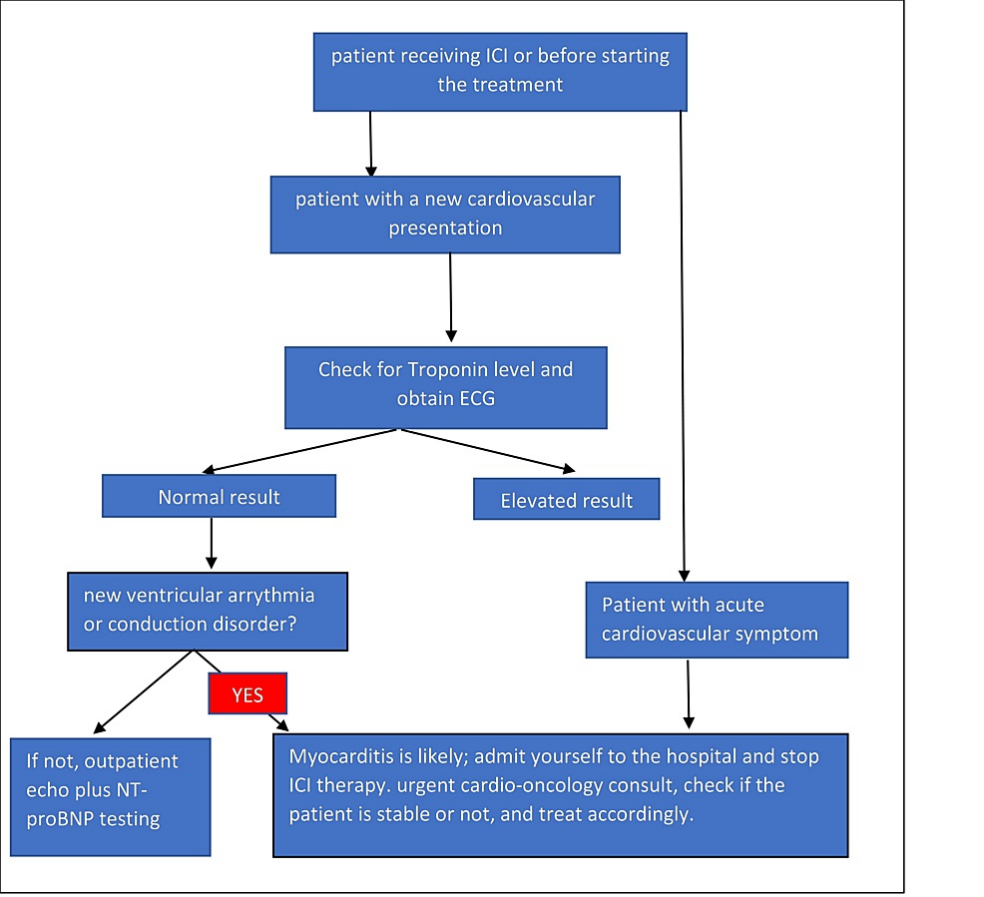
Workup and management of ICI-induced myocarditis. ICI = Immune checkpoint inhibitor, ECG = Electrocardiogram, NT-proBNP = N-Terminal pro-B-type natriuretic peptide [27].

A bedside instrument that can be used to diagnose myocarditis is an echocardiogram. Although echocardiography is a useful diagnostic tool, there are others, such as cardiac magnetic resonance (CMR), which is a promising diagnostic instrument for myocarditis. In CMR, the updated Lake Louis criteria for diagnosing myocarditis include T1 and T2 criteria. Gadolinium-enhanced CMR sequences reveal patchy myocardial hyperenhancement. The more invasive but gold-standard diagnosis of myocarditis is endomyocardial biopsy [22,31,32]. As mentioned in Table 4 in the study by Syed S. Mahmood et al., myocarditis isn't a common adverse effect of ICIs, but it can be fatal, and due to that, having a protocol to monitor the risk of myocarditis in patients receiving ICIs and treat them if indicated is necessary [27]. In Figure 3 by Syed S. Mahmood, you can see the algorithm for workup and management [27]. In the study conducted by Asuka Furukawa et al., the troponin levels of 126 patients treated with different ICIs revealed that 18 of them had elevated troponin levels; of these 18, 13 were clinically suspected of having myocarditis, but only 8 had symptoms [16]. According to the study, four participants exhibited mild symptoms, while the remaining four needed to stop their ICI therapy (Table 4). Although the results of the study showed a higher-than-expected rate of myocarditis, most of them had mild or no symptoms. This study showed that myocarditis mostly showed up early after ICI administration. However, many of the myocarditis susceptibility cases were clinically asymptomatic, so it is important to understand if they are pathologically significant or not. In this study, 69.2% of clinically suspected myocarditis cases were asymptomatic or modest. There are studies that show that despite the absence of clinical symptoms, autopsy reports on silent ICI myocarditis reveal patchy fibrosis and diffuse mononuclear cell infiltration of the myocardium [16]. In the study by Samantha N. Champion et al., which is in Table 4, ten cardiac tissues from patients with ICI-induced myocarditis were studied [25]. And they were classified into high-grade and low-grade based on their immunohistochemistry, such as CD3 count; the control group in this study was acute cellular allograft rejection. Lymphocyte-rich inflammation was seen in all myocarditis cases; however, only eight cases met the full Dallas criteria for myocarditis, and two other cases had borderline characteristics of myocarditis. In the study, three cases were classified as high-grade, and seven other cases were grouped as low-grade. These two classifications, low-grade and high-grade, depend on the density of the inflammation infiltration. A high grade is associated with more significant myocyte necrosis, which has a correlation with clinical outcomes. However, a larger number of studies are needed for this study [25].

Treatment

Myocarditis is difficult to treat, and there is no definitive protocol; however, the American Society of Clinical Oncology has suggested guidelines for ICI-induced myocarditis. According to this guideline, patients with grade 1 toxicity who are asymptomatic with elevated lab values suggesting myocarditis should immediately stop receiving the ICI permanently and start on high-dose prednisolone (1-2 mg/kg) [22]. Two different databases have shown that corticosteroids have a beneficial effect on myocarditis; however, there is no data in the case of failed corticosteroid treatment [26-28,33]. If the patient doesn't respond to high doses of prednisolone, they should start receiving methylprednisolone (1 gram/day) plus other immunosuppressants such as mycophenolate [6,22]. However, if the severity of myocarditis induced by ICIs is concerning, then treatment options can include anti-thymocyte globulin, IV immunoglobulins, or plasmapheresis [26]. A lack of precise guidelines regarding myocarditis induced by ICIs caused an increase in errors regarding the treatment; for example, a study regarding the use of infliximab to treat myocarditis showed that In patients with New York Heart Association (NYHA) class III or IV heart failure, using infliximab as an immunosuppressant for treating myocarditis-induced ICIs is contraindicated, and it's better to use tacrolimus with a high-dose corticosteroid [34]. In the study by Syed S. Mahmood et al., which can be found in Table 4, 35 patients from 8 distinct centers were diagnosed with ICI-induced myocarditis and compared to 105 patients who received ICI treatment without developing myocarditis. For the treatment, steroids were administered to 31 cases (89%) [27]. The mean time of steroid administration was approximately 21.4 hours, ranging from 1 to 60 hours; however, there was no difference in time of administration between the group with major adverse cardiac events (MACE) and the group without MACE. The median dose of methylprednisolone was 125 mg and ranged from 0 to 1000 mg. The MACE rate was lower in patients who began treatment with higher steroid doses. However, MACE occurred in two patients who received a methylprednisolone dose of 1000 mg. At the time of discharge, a higher dose of steroid was associated with a lower troponin level. Mycophenolate (in 2 patients), intravenous immunoglobulin (in 2 patients), anti-thymocyte globulin (in 1 patient), and infliximab (in 3 patients), and each of these groups had one patient who was successfully treated for myocarditis [27].

Limitation

This study has a number of limitations, including the use of unique and particular case reports of ICI-induced myocarditis, which enhances the likelihood of selection bias. Patients with previous heart problems or other conditions, such as autoimmune disease, which can affect the outcome of myocarditis, were not evaluated in this study. Only articles published between 2018 and 2023 were included, and those written in languages other than English were left out of the research. Future research must also examine the lack of defined treatment guidelines for ICI-induced myocarditis in order to reduce the risk of errors. Despite the limitations of this study, we assess the probable outcome of ICI myocarditis as well as how to approach evaluation, diagnosis, and treatment.

## Conclusions

Although ICI therapy is extremely beneficial for cancer patients, there is a risk of myocarditis; however, the majority of patients with ICI-induced myocarditis exhibit benign symptoms, some of which are concerningly fetal. Autoimmune reactions, cross-reactivity, and an increase in IL-17 levels are potential causes of ICI-related myocarditis. If myocarditis is suspected based on clinical symptoms, an electrocardiogram, and a troponin level, bedside echocardiography is the best way to confirm the diagnosis. However, additional tests, such as cardiac magnetic resonance imaging (CMR) and myocardial biopsy, can help confirm the diagnosis. Despite the lack of a definitive guideline, corticosteroids were found to be effective in the treatment of ICI-induced myocarditis. The lack of precise guidelines for the treatment of ICI-induced myocarditis, which can increase the error rate in patients with severe myocarditis, necessitates further research in the field. Further trials are required to determine the optimal time to initiate high-dose corticosteroids and the optimal combination of immune suppressants with corticosteroids.
